# Engeletin alleviates doxorubicin-induced cardiotoxicity via the AMPK pathway in mice

**DOI:** 10.3389/fphar.2026.1741741

**Published:** 2026-02-26

**Authors:** Xin Chen, Xing Zhong, Dan Luo, Qingning Huang, Pusong Tang, Lu Ye, Yuhua Lei, Rui Huang

**Affiliations:** 1 Cardiovascular Disease Center, The Central Hospital of Enshi Tujia and Miao Autonomous Prefecture, Enshi, Hubei, China; 2 Hubei Selenium and Human Health Institute, The Central Hospital of Enshi Tujia and Miao Autonomous Prefecture, Enshi, Hubei, China; 3 Hubei Provincial Key Lab of Selenium Resources and Bio Applications, Enshi, Hubei, China; 4 Cardiovascular Disease Center, The Central Hospital of Enshi Tujia and Miao Autonomous Prefecture, Hubei Minzu University, Enshi, Hubei, China

**Keywords:** AMPK pathway, apoptosis, cardiotoxicity, doxorubicin, Engeletin

## Abstract

**Background:**

The extensively employed antineoplastic drug doxorubicin (DOX) is constrained in clinical utilization on account of its severe cardiotoxicity, and there persists a dearth of protective agents against doxorubicin-induced cardiotoxicity (DIC). Engeletin (ENG) is a natural product endowed with multiple biological activities and has manifested significant protective effects in various diseases. This study purports to explore the protective effects of ENG in DIC and elucidate the underlying mechanisms.

**Methods:**

H9C2 cardiomyocytes and C57BL/6 mice were used to establish *in vitro* and *in vivo* models of DIC, and ENG was used for treatment. Cardiac function and structural changes in the mice were assessed by ultrasound, pathological section staining and transmission electron microscopy. Western blotting, Real-Time Quantitative PCR, immunofluorescence staining, enzyme-linked immunosorbent assay (ELISA), serum biochemical detection, TUNEL staining, dihydroethidium (DHE) assay, and flow cytometry were employed to evaluate apoptosis, autophagy, oxidative stress, inflammation, mitochondrial damage, ANP and BNP both *in vitro* and *in vivo*. An AMPK inhibitor Compound C was utilized to validate the effect of ENG on the AMPK pathway.

**Results:**

DOX diminished cardiac function and induced fibrosis in mice, resulting in significant cell apoptosis, oxidative stress, inflammation, autophagy dysregulation, and mitochondrial damage both *in vitro* and *in vivo*. Following ENG treatment, these conditions can be markedly ameliorated, especially in mitigating myocardial cell apoptosis, autophagy, and oxidative stress responses. It has been found that this effect is realized through the activation of the AMPK pathway. Moreover, utilization of an AMPK inhibitor CC impeded the protective effect of ENG on DIC.

**Conclusion:**

ENG has mitigated DIC through the activation of the AMPK pathway, thereby rendering it a potential drug for the prevention and treatment of DIC.

## 1 Introduction

Doxorubicin (DOX), a well-established anthracycline chemotherapeutic agent, is extensively utilized in clinical settings for the treatment of various malignancies (Go et al., 2018). However, its non-specific cytotoxicity is associated with significant cardiac damage, termed doxorubicin-induced cardiotoxicity (DIC), which can lead to irreversible cardiac dysfunction, potentially progressing to dilated cardiomyopathy and heart failure, and ultimately posing a life-threatening risk (Sheibani et al., 2022). The pathophysiology of DIC is complex, involving multiple processes such as apoptosis, autophagy, oxidative stress, pyroptosis, and ferroptosis (Rawat et al., 2021; Kong C. Y. et al., 2022; Christidi and Brunham, 2021). Despite substantial research efforts, effective preventive and therapeutic strategies for DIC remain limited. Currently, dexrazoxane is the sole DIC antagonist approved by the U.S. Food and Drug Administration (FDA) (Eneh and Lekkala, 2024; Monahan et al., 2021). However, its use is associated with significant adverse effects, including severe hepatic toxicity, myelosuppression, and secondary malignancies. Consequently, there is a pressing need for the development of more effective and safer cardioprotective agents to prevent and manage DIC.

Research into the cellular mechanisms and signaling pathways of DIC have confirmed that DOX can cause cardiomyocyte apoptosis, characterized by chromatin condensation, cell shrinkage, and the formation of apoptotic bodies (Qu et al., 2022). Additionally, it affects cardiomyocyte autophagic flux and alters the autophagosome to autolysosome ratio (Bartlett et al., 2017). Furthermore, evidence indicates that DOX treatment can result in excessive oxidative stress and mitochondrial damage, manifesting as lipid peroxidation, reactive oxygen species generation, and respiratory chain dysfunction. These processes ultimately lead to lipid peroxidation-dependent ferroptosis along with various other forms of regulated cell death (Christidi and Brunham, 2021; Kong C. Y. et al., 2022). Importantly, several cellular processes, including increased apoptosis, dysfunction of autophagy, mitochondrial damage, and formation of fibrosis, are all associated with adenosine monophosphate activated protein kinase (AMPK) (Lu et al., 2023). AMPK exerts anti-apoptotic effects by inhibiting molecular signaling pathways, enhances autophagy through ULK1 activation, and regulates mitochondrial metabolism via pgc1a signaling (Timm and Tyler, 2020). Numerous studies have demonstrated a close correlation between the activation of the AMPK signaling pathway and the treatment of DIC (Fan D. et al., 2023; Zhuo et al., 2023; Zhong Z. et al., 2023).

Engeletin (deoxydihydroquercetin-3-β-rhamnoside, ENG) is a naturally occurring flavonoid compound predominantly found in plants of the lily family and serves as a principal bioactive component in numerous traditional Chinese medicines (Zhong X. et al., 2023). Recent studies have identified ENG as possessing diverse biological activities, including antioxidant, anti-inflammatory, antibacterial, anti-tumor, and immunomodulatory effects (Feng et al., 2020; Zhao et al., 2020; Pushkala et al., 2022; Bai and Yin, 2020). Research indicates that ENG improves myocardial fibrosis and cardiac remodeling induced by isoproterenol (ISO) (Fang et al., 2023). Furthermore, ENG has demonstrated significant potential in the treatment of atherosclerosis (Wei et al., 2020). Several studies have shown that ENG can play a role in various diseases through multiple signaling pathways. Previous research has demonstrated that ENG stimulates adipocyte Browning by activating the β3-AR/AMPK signaling pathway (Kong L. et al., 2022). However, the protective effects and underlying mechanisms of ENG in DIC remains unkown.

At present, there are still no effective preventive and therapeutic measures for DIC, while ENG has demonstrated multiple cardiac protective effects. In this study, we aimed to investigate the role of ENG in DIC and clarify the mechanism and related signaling pathways of ENG in DIC through both *in vitro* and *in vivo* experiments so as to find new therapeutic targets and drugs for DIC.

## 2 Materials and methods

### 2.1 Main reagents and antibodies

Engeletin (HY-N0436), Doxorubicin (HY-15142A) were purchased from MedChemExpress (China). The antibodies used included Anti-beta Actin Rabbit pAb (Servicebio, 1:1000 dilution, #GB11001), Bax Rabbit pAb (ABclonal, 1:1000 dilution, #A12009), Bcl2 Mouse Monoclonal Antibody (Proteintech, 1:1000 dilution, #66799-1-Ig), Beclin 1 Antibody (Abmart, 1:1000 dilution, #T55092), LC3B Antibody (Abmart, 1:1000 dilution, #T55992), TNF Alpha antibody (Proteintech, 1:500 dilution, #60291-1-Ig), AMPK alpha 1 Antibody (Abmart, 1:1000 dilution, #T55326), Phospho-AMPK alpha (T172) Antibody (Abmart, 1:1000 dilution, #T55608), BNP Antibody (Abmart, 1:1000 dilution, #PK54192), ANP Antibody (Abmart, 1:1000 dilution, #T57175), HRP-conjugated Goat Anti-Mouse IgG (H + L) (Proteintech, 1:5000 dilution, #SA00001-1) and HRP-conjugated Goat Anti-Rabbit IgG (H + L) (Proteintech, 1:5000 dilution, #SA00001-2).

### 2.2 Animal experiments

The animal feeding and experimental procedures were conducted in accordance with the guidelines outlined in the Guide for the Care and Use of Laboratory Animals of the National Institutes of Health (NIH), and were approved by the Animal Ethics Committee of The Central Hospital of Enshi Tujia and Miao Autonomous Prefecture. Male C57BL/6 mice were purchased from the Experimental Animal Center of China Three Gorges University. Prior to the commencement of the experiments, the mice underwent a one-week acclimatization period. All mice were raised under a 12-h light/dark cycle in a pathogen-free environment, with controlled conditions (temperature: 20 °C ± 2 °C; humidity: 40%–60%), and were provided with *ad libitum* access to water and food. Upon conclusion of the experiment, the mice were anesthetized using 5% isoflurane and subsequently euthanized via cervical dislocation.

To investigate the effects of ENG *in vivo*, we established a chronic DIC mouse model by intraperitoneal injection of DOX (5 mg/kg/w for 5 weeks) (Wu et al., 2024) and intervened with intraperitoneal injection of ENG (20 mg/kg/d for 2 weeks) (Fang et al., 2023). Twenty 8-week-old male mice were randomly divided into four groups (five per group): (1) Control group: intraperitoneal injection of an equal volume of saline. (2) ENG group: 20 mg/kg/d of ENG for 2 weeks. (3) DOX group: 5 mg/kg/w of DOX for 5 weeks. (4) DOX + ENG group: 20 mg/kg/d of ENG for 2 weeks, and 5 mg/kg/w of DOX for 5 weeks. At the conclusion of week 6, echocardiographic assessments were conducted, followed by the collection of blood samples and heart tissue from the mice.

### 2.3 Echocardiography

To assess the cardiac function of mice, after anesthesia and weighing, the left ventricular end-diastolic diameter (LVEDd), left ventricular end-systolic diameter (LVESd), left ventricular ejection fraction (LVEF) and left ventricular fractional shortening (LVFS) were recorded using a Vinno6 echocardiography system (Vinno Technology, Suzhou, Jiangsu, China) operating at 15.0 MHz.

### 2.4 Biochemical detection

After the mice were executed, peripheral blood was collected in an EDTA tube and centrifuged at 1500 rpm for 15 min at 4 °C. Serum was extracted for the determination of malondialdehyde (MDA) levels using an MDA assay kit (#G4300, Servicebio), following the manufacturer’s instructions.

### 2.5 Histological assessment

The isolated mice heart tissues were fixed with 4% paraformaldehyde, followed by dehydrated, embedded, and cut into 4-μm thick sections. Hematoxylin and eosin (HE) staining was performed on the sections to examine cardiac histomorphology. Masson staining was used to assess myocardial fibrosis. Apoptosis was detected using TUNEL staining. Additionally, lipid peroxidation was assessed by staining heart sections with 4-hydroxynonenal (4HNE), and images were captured using fluorescence microscopy (Qu et al., 2022).

### 2.6 Transmission electron microscope

Fresh heart tissue was cut into small pieces (1–3 mm^3^), fixed with 2.5% glutaraldehyde at room temperature in the dark for 2 h, and then stored at 4 °C. Next, dehydration and embedding were performed. Ultrathin sections of tissue were made with a diamond knife and stained with uranyl acetate and lead citrate (Zhuo et al., 2023). Transmission electron microscopy was employed to capture images of the sections.

### 2.7 Cell viability assay

Cell viability was measured using a cell counting kit-8 (CCK-8, #G4103, Servicebio). H9C2 cells (Wuhan Zishan Biotechnology Co., Ltd.) were seeded at a density of 1 × 10^4^/well in 96-well plates and cultured for 24 h. Next, the cells were treated with different concentrations of ENG. Then, according to the kit instructions, 10 μL of CCK8 reagent was added to each group and incubated for 2 h. Absorbance was subsequently measured at 450 nm using a microplate reader to evaluate cell viability (Lu et al., 2023).

### 2.8 Cell culture and treatment

H9C2 cells were cultured in DMEM/F12 medium supplemented with 10% fetal bovine serum, 1% penicillin, and 1% streptomycin. The cells were maintained in a humidified atmosphere containing 5% CO2 at 37 °C. When the cells reached 80% confluency, we performed cell passaging and processing. To investigate the role of ENG in the *in vitro* model of DIC, we established this model using H9C2 cells treated with 1 µM DOX for 24 h (Qiu et al., 2024), and employed ENG at a concentration of 80 µM for 24 h (Li et al., 2022). as an interventional therapy. H9C2 cells were randomly assigned to four groups: the Control group (treated with an equal volume of solvent for 24 h), the DOX group (exposed to 1 µM DOX for 24 h), the ENG group (administered 80 µM ENG for 24 h), and the DOX + ENG group (pre-treated with 80 µM ENG for 2 h, followed by co-treatment with 1 µM DOX for 24 h). To assess the AMPK pathway, H9C2 cells were pre-treated with the specific AMPK inhibitor Compound C (CC) at a concentration of 10 µM for 18 h (Zhou et al., 2022), subsequently treated with ENG for 2 h and then co-incubated with 1 µM DOX for 24 h (DOX + ENG + CC group).

### 2.9 Measurement of reactive oxygen species

Dihydroethidium (DHE, #G1904, Servicebio) was employed to measure reactive oxygen species (ROS) in H9C2 cells. In accordance with the manufacturer’s protocol, a 5 μM DHE detection working solution was added to the treated cells and incubated in a dark environment at 37 °C for 30 min within a cell culture incubator (Zhuo et al., 2023). Fluorescence images were subsequently acquired, and fluorescence intensity was evaluated using a fluorescence microscope.

### 2.10 TUNEL staining

Following the fixation and permeabilization of H9C2 cells cultured in 6-well plates, the apoptosis of cells was detected using an *in situ* apoptosis detection kit. Images were obtained using an Olympus FV 1000 laser scanning confocal microscope (Olympus, Japan).

### 2.11 Immunofluorescence staining

Following drug treatment, H9C2 cells cultured in 6-well plates were fixed, permeabilized, and subjected to blocking of non-specific binding according to the protocol provided by the immunofluorescence (IF) staining kit. Subsequently, the cells were incubated overnight at 4 °C with a specific monoclonal antibody against Beclin 1 (Abmart, 1:1000 dilution, #T55092) and TNF-α (Proteintech, 1:500 dilution, #60291-1-Ig). After washing three times with phosphate-buffered saline (PBS), the cells were then incubated for 1 h at room temperature with a red fluorescent-labeled secondary antibody. Finally, DAPI was employed to stain the cell nuclei for fluorescence signal imaging (Lu et al., 2023).

### 2.12 Flow cytometry

The Annexin V-FITC Apoptosis Detection Kit was employed to quantify the apoptosis rate. Cardiomyocytes subjected to various treatments were harvested, rinsed twice with cold PBS, and subsequently incubated with annexin V-FITC and propidium iodide (PI) for 15 min at ambient temperature in the absence of light. Apoptosis was ultimately assessed using a flow cytometer (Zhong Z. et al., 2023).

### 2.13 Western blot

The myocardial tissues and H9C2 cells were collected and homogenized in RIPA buffer supplemented with protease and phosphatase inhibitors, followed by a 25-min lysis on ice to extract total proteins. The supernatant was collected after centrifugation at 12,000 rpm for 30 min at 4 °C. Protein concentration was determined using the BCA protein quantification kit (#AR0146, BOSTER, Wuhan, China). Samples were diluted with loading buffer and denatured by heating at 95 °C for 5 min. Equal amounts of protein samples (30 μg/well) were subjected to electrophoretic separation on a 12% sodium dodecyl sulfate-polyacrylamide gel and subsequently transferred onto a 0.45 μm PVDF membrane. The membranes were blocked with 5% skimmed milk at room temperature for 1 hour before being incubated overnight with specific primary antibodies at 4 °C. After washing, the membranes were incubated with secondary antibodies at room temperature for 2 h (Fan D. et al., 2023). Protein bands were then visualized using a highly sensitive ECL chemiluminescence kit (#BMU102-CN, Abbkine, China) in the ChemiDoc XRS + system (Bio-Rad). Finally, quantitative analysis was conducted using ImageJ software (National Institutes of Health, United States).

### 2.14 Real-Time Quantitative PCR

RNA was extracted from H9C2 cells utilizing the RNA Rapid Extraction Kit (RNO7, Aidlab, China) in accordance with the manufacturer’s instructions. 1 μg of RNA was reverse transcribed into cDNA using the cDNA Reverse Transcription Kit in a 20 μL reaction system. Real-Time Quantitative Polymerase Chain Reaction (RT-qPCR) was performed using a PCR detection system (7500fast, Applied Biosystems, Foster City, CA, United States) to detect the mRNA expression level (Fan D. et al., 2023).

### 2.15 Enzyme-linked immunosorbent assay (ELISA)

At the conclusion of the treatment, the mice were euthanized, and peripheral blood samples were collected in tubes containing EDTA. The blood samples were centrifuged at 1500 rpm for 15 min at 4 °C to isolate serum, which was subsequently used to measure B-type Natriuretic Peptide (BNP) levels, a recognized biomarker of myocardial injury. BNP levels in mouse serum were quantified using an ELISA kit (#AB-K554102, Abmart), following the manufacturer’s recommended protocol (Zhuo et al., 2023).

### 2.16 Statistical analysis

Statistical analyses were conducted using GraphPad Prism version 9.5 (GraphPad Software, San Diego, CA, United States). Quantitative data are presented as mean ± standard error of the mean (SEM). A one-way analysis of variance (ANOVA) was employed to assess differences among multiple groups, followed by Tukey’s *post hoc* test. *P* < 0.05 was considered statistically significant for differences.

## 3 Results

### 3.1 ENG alleviates DOX-induced cardiotoxicity in mice

**FIGURE 1:**
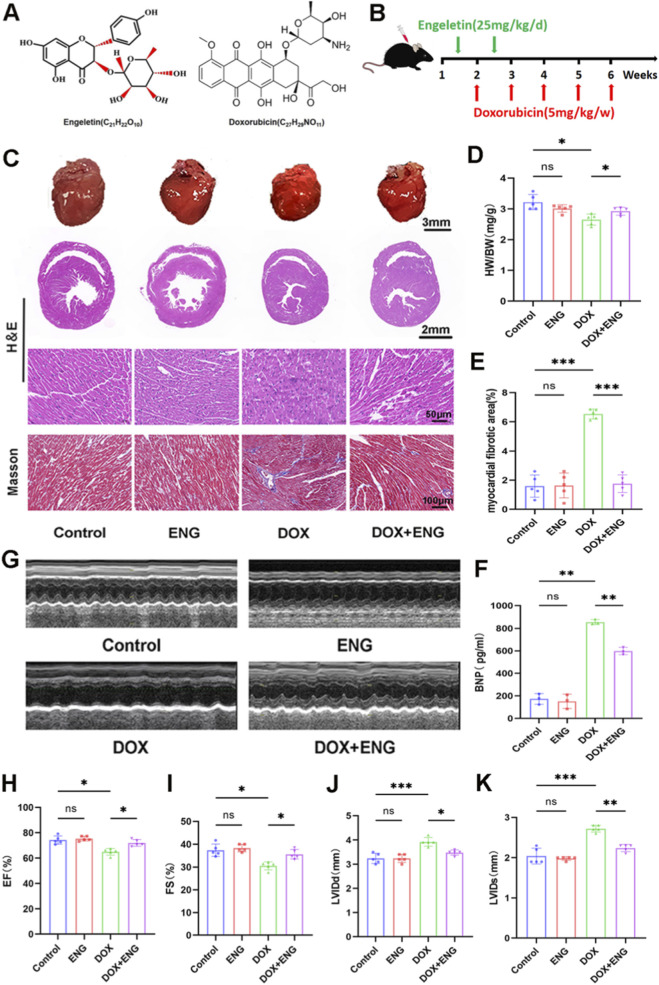
ENG alleviates DOX-induced cardiotoxicity in mice. **(A)** Molecular structure of ENG and DOX. **(B)** Flow chart of the experimental protocol *in vivo*. **(C)** Representative images of gross heart morphology, HE staining and Masson’s staining of mice in each group (n = 5). **(D)** The ratio of heart weight to body weight (HW/BW) (n = 5). **(E)** The quantification of myocardial fibrosis area (n = 5). **(F)** Expression of BNP in the serum of each group of mice (n = 3). **(G)** Representative image of an M-mode echocardiogram (n = 5). **(H–K)** Analyses of LVEF, LVFS, LVEDd and LVESd (n = 5). Statistical analysis was performed using one-way ANOVA, followed by Tukey’s *post hoc* test. *P < 0.05, **P < 0.01, ***P < 0.001.

To investigate the potential role of ENG in DIC, we established a mice model of chronic myocardial injury by intraperitoneal injection of DOX (5 mg/kg) once a week for 5 weeks. Prior to DOX treatment, mice were treated with ENG (20 mg/kg) intraperitoneally once a day for 14 days (Figures 1A,B). The mice treated with DOX exhibited significant weight loss and a decrease in heart volume, which was mitigated by ENG treatment. HE and Mosson staining were used to evaluate cardiac tissue structure, and the results showed that myocardial morphology was disordered, and perivascular and interstitial fibrosis was increased in DOX group. However, these manifestations were significantly improved in mice treated with ENG (Figures 1C–E). The serum BNP concentration, as measured by the ELISA kit, demonstrated that the DOX group exhibited a significantly higher BNP level compared to the control group, and ENG treatment markedly ameliorated this elevation (Figure 1F). M-mode echocardiography was employed to evaluate cardiac function, and the findings indicated DOX-treated mice had significant cardiac function reduction. LVEF, LVFS, LVEDd, and LVESd of mice were significantly restored after ENG treatment (Figures 1G–K). No significant differences were observed in the aforementioned results between the control group and the ENG group.

### 3.2 ENG improve DOX-induced apoptosis and autophagy in mice

**FIGURE 2:**
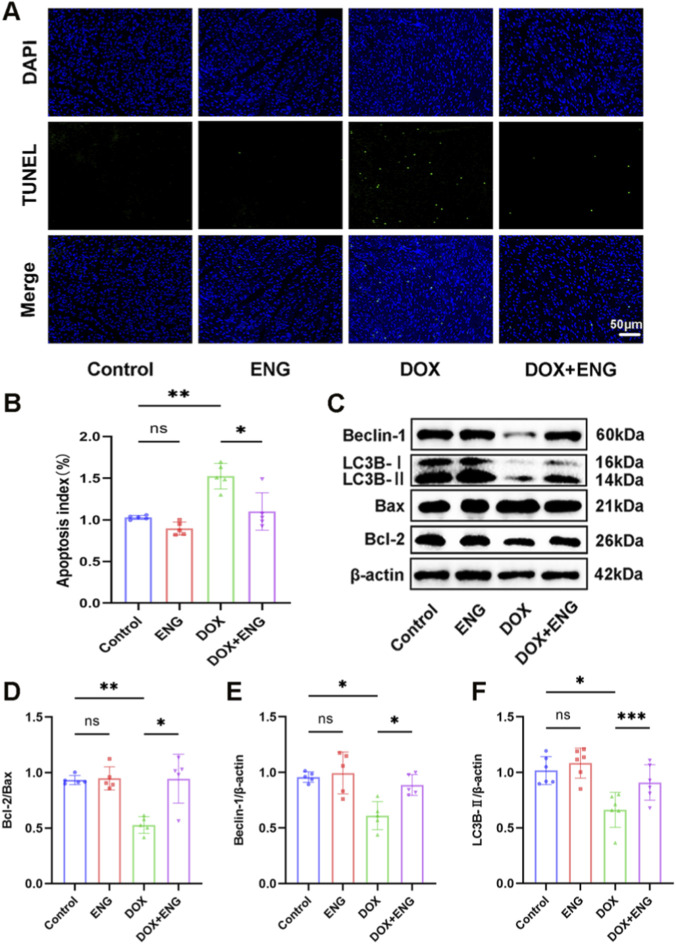
ENG improve DOX-induced apoptosis and autophagy in mice. **(A,B)** Representative images and quantitative analysis of TUNEL staining of mice myocardium (n = 5). **(C–F)** Western blotting results of Beclin-1, LC3B, Bax and Bcl-2 in mice cardiac tissue and quantitative analysis (n = 5). Statistical analysis was performed using one-way ANOVA, followed by Tukey’s *post hoc* test. *P < 0.05, **P < 0.01, ***P < 0.001.

Several studies have demonstrated that DOX can exacerbate cardiomyocyte apoptosis and impede autophagy (Zhang et al., 2023; Fan X. et al., 2023). To investigate the potential of ENG in mitigating these effects, we assessed key markers of apoptosis and autophagy in cardiac tissues derived from DOX and ENG-treated mice. TUNEL staining of myocardial tissue revealed a significant elevation in apoptotic cells among the mice administered DOX, whereas a substantial reduction in apoptotic cells was observed in the mice treated with ENG (Figures 2A,B). Western blot analysis indicated that ENG mitigated the upregulation of Bax and downregulation of Bcl-2 induced by DOX, while also inhibiting the downregulation of beclin-1 and LC3B caused by DOX treatment (Figures 2C–F). No significant differences were detected between the control and ENG groups in these parameters. These findings substantiate that ENG effectively inhibits DOX-induced apoptosis and restores autophagic function in myocardial tissues of mice.

### 3.3 ENG mitigated DOX-triggered oxidative stress and improved of mitochondrial function

**FIGURE 3:**
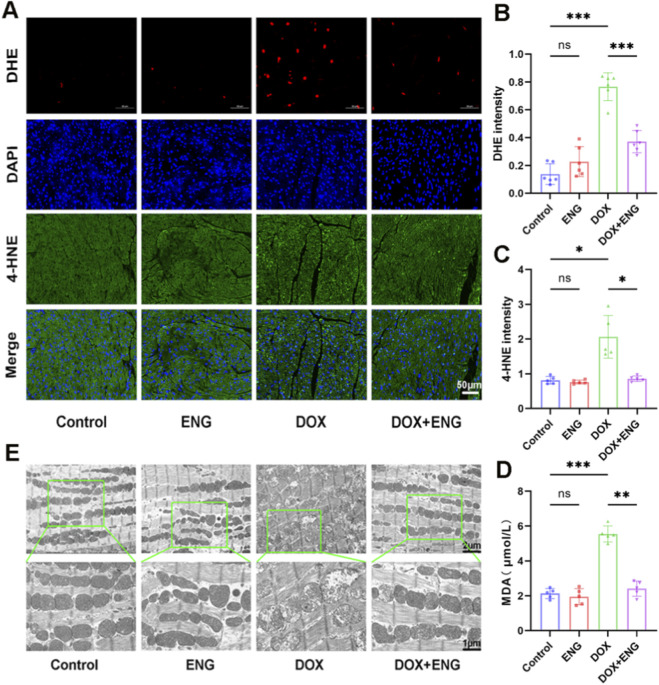
ENG mitigated DOX-triggered oxidative stress and improved of mitochondrial function. **(A–C)** Representative images and quantitative analysis of DHE staining of cardiomyocytes (n = 6) and 4-HNE staining of mice cardiac tissue (n = 5). **(D)** MDA level in mice serum (n = 5). **(E)** Representative images of mitochondria from the myocardium of mice in each group. Statistical analysis was performed using one-way ANOVA, followed by Tukey’s *post hoc* test. *P < 0.05, **P < 0.01, ***P < 0.001.

Previous studies have implicated oxidative stress in the development of DIC (Chen et al., 2024). In this study, we evaluated several main indicators of oxidative stress, including MDA levels, 4-HNE levels and DHE staining. Firstly, DHE staining of H9C2 cells showed that The levels of ROS were significantly increased in the DOX group compared to the control and ENG groups, whereas ROS were significantly decreased in the DOX + ENG group compared with the DOX group (Figures 3A,B). We further conducted 4-HNE staining on myocardial tissue from various treatment groups, observing minimal green fluorescence intensity in the control and ENG groups. In contrast, the DOX group exhibited a marked increase in green fluorescence intensity, which was significantly diminished in the DOX + ENG group (Figures 3A–C). Additionally, serum MDA levels were measured across different treatment groups, revealing a significantly higher concentration in the DOX group compared to the other groups, with statistical significance (Figure 3D). The results suggested that ENG alleviated the oxidative stress response induced by DOX.

To assess the protective effect of ENG on mitochondrial function, we conducted a transmission electron microscope examination of myocardial tissue to observe mitochondrial morphology. Electron microscopy revealed mitochondrial damage, including matrix swelling, cristae fragmentation, breakage, and disappearance in the DOX group. Conversely, this phenomenon was reversed in the DOX + ENG group (Figure 3E). There was no obvious mitochondrial damage in the control and ENG groups. The findings indicate that ENG reversed DOX-induced mitochondrial damage in cardiomyocytes.

### 3.4 ENG attenuated DOX-induced cardiomyocyte apoptosis and functional damage

**FIGURE 4:**
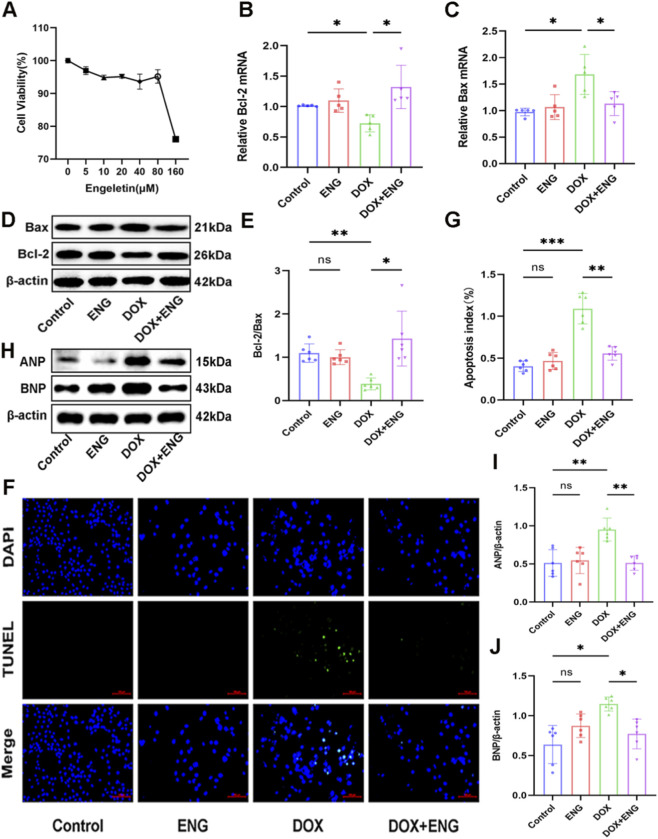
ENG attenuated DOX-induced cardiomyocyte apoptosis. **(A)** Relative cell viability of ENG. **(B,C)** Relative cardiomyocyte mRNA levels of Bcl-2 and Bax (n = 5). **(D,E)** Western blotting results of Bax and Bcl-2 in cardiomyocytes and quantitative analysis (n = 6). **(F,G)** Representative images and quantitative analysis of TUNEL staining of cardiomyocytes (n = 6). **(H–J)** Western blotting results of ANP and BNP in cardiomyocytes and quantitative analysis (n = 6). Statistical analysis was performed using one-way ANOVA, followed by Tukey’s *post hoc* test. *P < 0.05, **P < 0.01, ***P < 0.001.

Prior to conducting experiments on H9C2 cells, cardiomyocytes were exposed to varying concentrations of ENG (0, 5, 10, 20, 40, 80, and 160 µM), and cell viability was assessed by CCK-8 kit (Figure 4A). The results showed that ENG at low concentration had no significant cytotoxic effect on cardiac myocytes. Based on the results of previous studies (Li et al., 2022) and our cell viability assay, ENG concentration of 80 µM was selected to treat cardiomyocytes in the subsequent investigations.

H9C2 cells were then categorized into different experimental groups for comparative analysis using RT-qPCR, Western blot analysis and TUNEL staining. In the DOX group, the mRNA expression levels of the pro-apoptotic marker Bax were significantly elevated compared to those in the control and ENG group. ENG treatment ameliorated the DOX-induced upregulation of Bax and downregulation of Bcl-2 (Figures 4B,C). Western blot analysis corroborated these findings, showing an upregulation of Bax and a downregulation of Bcl-2 in the DOX group, which were reversed in the DOX + ENG group. No significant differences in expression were observed between the control and ENG groups (Figures 4D,E). In the TUNEL staining of cardiomyocytes, we observed a significantly higher number of apoptotic cells in the DOX group than other groups, and no significant difference was observed between the control and ENG groups (Figures 4F,G). These results indicated that ENG significantly attenuated DOX-induced cardiomyocyte apoptosis *in vitro*. Western blot analysis of the aforementioned cell groups revealed that the expression levels of ANP and BNP were elevated in the DOX group, and these levels were markedly reduced following ENG treatment. Furthermore, no significant difference in expression was observed between the control and ENG groups, indicating that ENG effectively alleviated DOX-induced functional damage in cardiomyocytes (Figures 4H–J).

### 3.5 ENG mitigated DOX-triggered inflammatory and improved autophagy in cardiomyocytes

**FIGURE 5:**
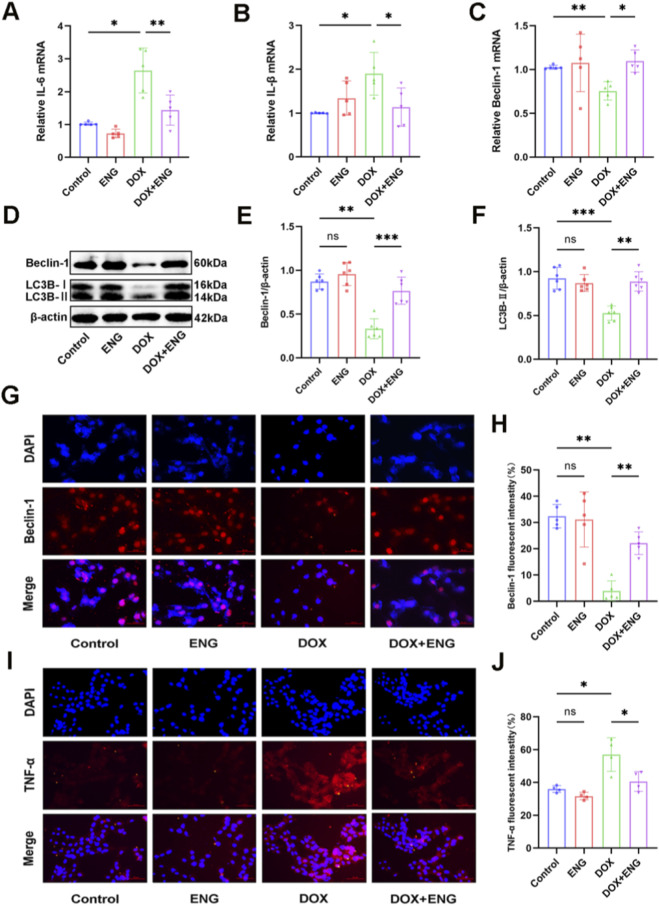
ENG mitigated DOX-triggered inflammatory and improved autophagy in cardiomyocytes. **(A–C)** Relative cardiomyocyte mRNA levels of IL-6, IL-β and Beclin-1 (n = 5). **(D–F)** Western blotting results of Beclin-1 and LC3B in cardiomyocytes and quantitative analysis (n = 6). **(G,H)** Representative images and quantitative analysis of Beclin-1 immunofluorescence staining in cardiomyocytes (n = 5). **(I,J)** Representative images and quantitative analysis of TNF-α immunofluorescence staining in cardiomyocytes (n = 4). Statistical analysis was performed using one-way ANOVA, followed by Tukey’s *post hoc* test. *P < 0.05, **P < 0.01, ***P < 0.001.

To verify the protective effect of ENG in DOX-induced inflammatory responses in cardiomyocytes, we conducted *in vitro* experiments. The RT-qPCR results revealed a marked upregulation in mRNA expression levels of IL-6 and IL-1β in the DOX group, while the control group and ENG groups exhibited low expression levels and not significantly different between the two groups. Treatment with ENG effectively reduced DOX-induced inflammatory mRNA expression (Figures 5A,B). Furthermore, TNF-α immunofluorescence staining in cardiomyocytes corroborated the RT-qPCR findings (Figures 5I,J), thereby substantiating that ENG alleviates the inflammatory response induced by DOX in cardiomyocytes.

At the same time, we evaluated the protective effect of ENG on cardiomyocyte autophagy function through *in vitro* experiments. First, we determined the Beclin-1 mRNA expression levels of different groups, and the results suggest that autophagy function was impaired in the DOX group, whereas cardiomyocyte autophagy was restored after ENG treatment, and there was no significant difference in expression between the control and ENG groups (Figure 5C). Secondly, Western blot analysis was employed to detect the autophagy markers Beclin-1 and LC3B. The expression of both Beclin-1 and LC3B was significantly reduced in the DOX group but upregulated in the DOX + ENG group (Figures 5D–F). Finally, immunofluorescence staining for Beclin-1 was utilized to validate cardiomyocyte autophagy among different groups, demonstrating that ENG treatment could rectify the DOX-induced decline in autophagic function (Figures 5G,H).

### 3.6 ENG alleviates DOX-induced cardiomyocyte injury through the AMPK pathway

**FIGURE 6:**
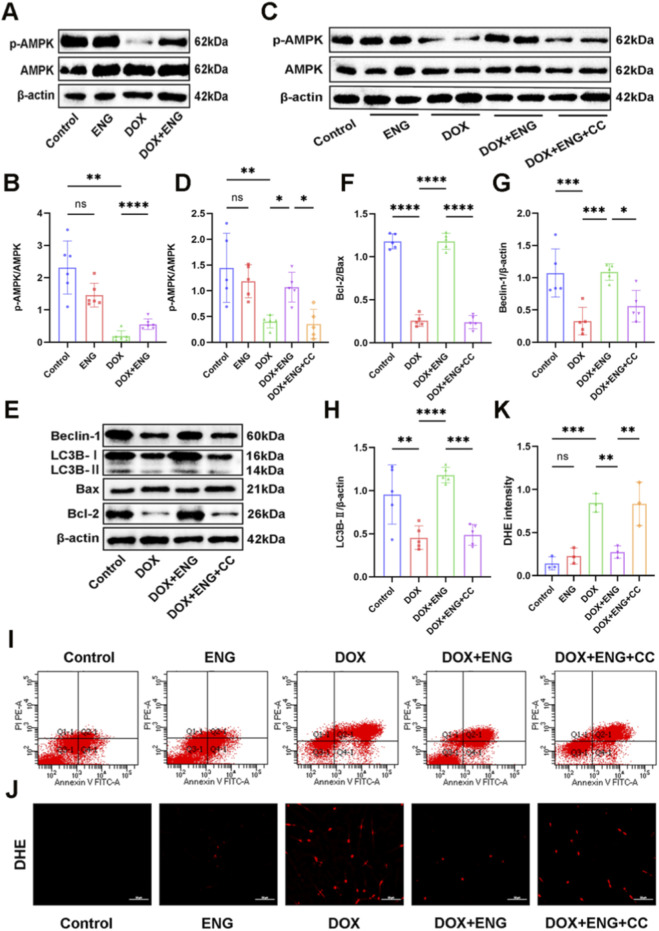
ENG alleviates DOX-induced cardiomyocyte injury through the AMPK pathway. **(A,B)** Western blotting results and quantitative analysis of p-AMPK and AMPK in mice cardiac tissue (n = 6). **(C,D)** Western blotting results and quantitative analysis of p-AMPK and AMPK in cardiomyocytes (n = 5). **(E–H)** Western blotting results of Beclin-1, LC3B, Bax and Bcl-2 in cardiomyocytes and quantitative analysis (n = 5). **(I)** Flow cytometry of cardiomyocyte apoptosis in each group. **(J,K)** Representative images and quantitative analysis of DHE staining of cardiomyocytes (n = 6). Statistical analysis was performed using one-way ANOVA, followed by Tukey’s *post hoc* test. *P < 0.05, **P < 0.01, ***P < 0.001.

Previous studies have demonstrated that DOX can cause cardiotoxicity through the AMPK pathway and that ENG may play a role in other diseases through the AMPK pathway (Zhuo et al., 2023; Zhong Z. et al., 2023; Kong L. et al., 2022). In our study, Western blot analysis revealed a significantly reduced expression of phosphorylated AMPK (p-AMPK) in the DOX group compared to other groups. No significant difference in p-AMPK expression was observed between the control group and the ENG group. However, p-AMPK expression was upregulated in the DOX + ENG group, and the p-AMPK/AMPK ratio was notably lower in the DOX group compared to the DOX + ENG group (Figures 6A,B). To further verify the mechanism, when cardiomyocytes treated with both DOX and ENG were exposed to Compound C (CC), a specific inhibitor of AMPK, there was a downregulation of p-AMPK expression suggesting that CC blocked activation of the AMPK signaling pathway by ENG (Figures 6C,D).

Subsequently, Western blot analysis was used to detect the apoptosis and autophagy of cardiomyocytes. ENG ameliorated DOX-induced apoptosis and impaired autophagy, whereas the addition of CC inhibited this protective effect of ENG (Figures 6E–H). Apoptosis in cardiomyocytes across different groups was evaluated using flow cytometry, revealing a significantly higher number of apoptotic cells in the DOX group compared to both the control and ENG groups. Furthermore, the DOX + ENG + CC group exhibited a significantly higher number of apoptotic cells than the DOX + ENG group (Figure 6I). Finally, we detected oxidative stress in each group by DHE staining. Consistent with the previous results, oxidative stress was evident in the DOX group, while it was alleviated in the DOX + ENG group, but the addition of CC significantly increased oxidative stress in cardiomyocytes (Figures 6J,K). These results indicated that CC blocked the effects of ENG in ameliorating apoptosis, autophagy and oxidative stress in cardiomyocytes by inhibiting the AMPK pathway, and verified that ENG alleviated DIC by activating AMPK signaling pathway.

## 4 Discussions

**FIGURE 7:**
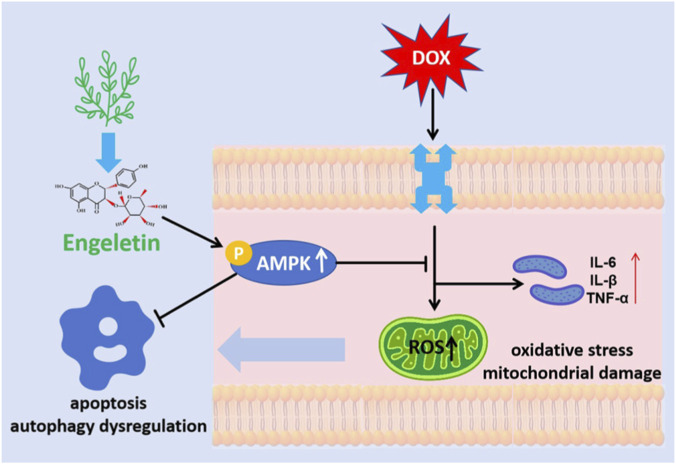
Revealing the mechanistic pathways underlying the cardioprotective effects of ENG against DOX-induced cardiotoxicity.

The results of this study demonstrated that ENG exerted significant protective effects on DIC both *in vitro* and *in vivo*. These effects were manifested by the inhibition of apoptosis and oxidative stress, attenuation of inflammatory response, enhancement of autophagy, restoration of mitochondrial damage, and protection of cardiomyocyte function. Notably, our research identified that these protective mechanisms are facilitated through the activation of the AMPK pathway, and that inhibition of this pathway can negate the cardioprotective effects of ENG (Figure 7).

DOX has emerged as a pivotal agent in the chemotherapy of malignancies. Nevertheless, DIC remains a grave adverse effect with limited preventive and therapeutic approaches (Chen et al., 2022). Despite continuous investigation into DIC, its etiology and pathogenesis are intricate, leaving a dearth of effective strategies. Consequently, there is an imperative need to identify potential targets and pharmacological interventions. Numerous previous studies have identified ENG, a class of polyphenolic natural products, exhibits diverse biological activities and holds potential pharmacological protective effects in various diseases, including antioxidant, anti-inflammatory, and immune regulatory properties (Zhong X. et al., 2023). ENG has been shown to exert neuroprotective effects, attenuating ischaemia/reperfusion injury and Alzheimer’s disease (Xu et al., 2023). Furthermore, ENG functions as a natural aldose reductase (AR) inhibitor, exerting an antioxidant effect and synthesises a variety of anti-inflammatory mediators to inhibit the inflammatory response (Zhao et al., 2020). In this study, ENG exhibited remarkable antioxidant stress and anti - inflammatory effects on DOX - induced myocardial injury.

The primary mechanism underlying DIC is cardiomyocyte apoptosis, a prominent form of programmed cell death. DOX can permeabilize the mitochondrial outer membrane, allowing the diffusion of various proteins into the cytoplasm and subsequently activating the endogenous apoptotic pathway (Christidi and Brunham, 2021). DOX induces excessive generation of reactive ROS and a significant elevation in lipid peroxidation levels, leading to oxidative stress. This oxidative stress can activate heat shock factor 1 (HSF-1), which in turn produces pro-apoptotic factors that contribute to cardiomyocyte death (Rawat et al., 2021). The strong affinity between DOX and cardiolipin enables its accumulation within mitochondria, triggering oxidative stress that impairs mitochondrial function and initiates apoptosis (Wu et al., 2022). Additionally, DOX disrupts genes involved in autophagy while also impairing autophagic flux and inhibiting lysosomal acidification in cardiomyocytes. These effects promote ROS production and cellular demise (Christidi and Brunham, 2021). Simultaneously, DOX activates the NLRP3 inflammasome within cardiomyocytes, leading to a significant release of pro-inflammatory cytokines that further stimulate cardiomyocyte apoptosis. Consequently, this process exacerbates adverse cardiac remodeling and contributes to heart failure development (Shi et al., 2023). Our previous review studies have elucidated that ENG possesses multiple significant biological activities (Zhong X. et al., 2023). Studies have demonstrated that ENG exhibits antioxidant and anti-inflammatory properties in a mouse model of ISO-induced myocardial fibrosis, leading to reduced generation of ROS and MDA activity (Pushkala et al., 2022). Furthermore, previous research has indicated that ENG inhibits the TNF-α-induced upregulation of inflammatory mediators and apoptosis of NP cells (Li et al., 2022)^.^ Additionally, ENG has been shown to activate autophagy by stimulating the expression of autophagy markers and autophagy-related genes in lung cancer cells (Liu et al., 2020). Consistent with prior findings, ENG exerts anti-apoptotic, anti-oxidative stress, anti-inflammatory effects while promoting repair of autophagy and mitochondrial function damage, thereby contributing to the protection of cardiomyocyte function.

Numerous studies have established a significant association between the mechanism of DIC and AMPK. Activation of AMPK has been shown to reduce apoptosis by inhibiting the mTOR signaling pathway, enhance autophagy through the ULK1, improve mitochondrial metabolism via PGC1α signaling, and mitigate fibrosis by suppressing the TGFβ signaling pathway (Lu et al., 2023; Timm and Tyler, 2020). Various pharmacological agents have demonstrated anti-DIC effects through the activation of the AMPK pathway (Zhuo et al., 2023; Singh et al., 2022). ENG has also been discovered to exert protective effects by activating the β3-AR/AMPK signaling pathway (Kong L. et al., 2022). This study further proved that ENG can activate the AMPK signaling pathway to alleviate DIC, and the AMPK inhibitor CC can impede the beneficial effects of ENG on DIC.

Despite the substantial protective effect of ENG in DIC demonstrated in this study, certain limitations must be acknowledged. Firstly, the study did not assess whether ENG influences the anti-tumor efficacy of Dox. The complexity inherent in tumor treatment poses challenges in evaluating the impact of ENG across all DOX-sensitive tumor models. Secondly, the *in vitro* experiments utilized H9C2 cells instead of primary cardiomyocytes from mice, which may not fully replicate the physiological conditions present in mice. Lastly, the investigation of ENG’s role in the AMPK pathway was confined to the cellular level using the AMPK inhibitor CC, without corroboration through the use of heart-specific AMPK gene-knockout mice.

## 5 Conclusion

This study clarifies for the first time that ENG alleviates cardiomyocyte apoptosis, oxidative stress, autophagy dysfunction, mitochondrial damage, and inflammatory response caused by DOX through the activation of the AMPK pathway, thereby avoiding myocardial fibrosis and heart failure. These findings suggest that ENG may be a natural product with significant potential for application in the prevention and treatment of DIC.

## Data Availability

The original contributions presented in the study are included in the article/Supplementary Material, further inquiries can be directed to the corresponding authors.

## Glossary

DOX: doxorubicin
DIC: doxorubicin-induced cardiotoxicity
ENG: Engeletin
FDA: Food and Drug Administration
AMPK: adenosine monophosphate activated protein kinase
ULK1: Unc-51-like autophagy activating kinase 1
ISO: isoproterenol
NIH: National Institutes of Health
LVEDd: left ventricular end-diastolic diameter
LVESd: left ventricular end-systolic diameter
LVEF: left ventricular ejection fraction
LVFS: left ventricular fractional shortening
MDA: malondialdehyde
HE: Hematoxylin and eosin
4HNE: 4-hydroxynonenal
CCK-8: cell counting kit-8
CC: Compound C
ROS: reactive oxygen species
IF: immunofluorescence
DHE: Dihydroethidium
PBS: phosphate-buffered saline
TNF-α: Tumour necrosis factor-alpha
PI: propidium iodide
RT-qPCR: Real-Time Quantitative Polymerase Chain Reaction
ELISA: Enzyme-linked immunosorbent assay
BNP: B-type Natriuretic Peptide
SEM: mean ± standard error of the mean
ANOVA: A one-way analysis of variance
LC3B: light chain 3B
AR: aldose reductase
HSF-1: heat shock factor 1
NLRP3: NLR family pyrin domain-containing 3
TGF-β: Transforming growth factor beta
mTOR: mammalian target of rapamycin.

## References

[B1] Bai H. Yin H. (2020). Engeletin suppresses cervical carcinogenesis *in vitro* and *in vivo* by reducing NF-κB-dependent signaling. Biochem. Biophys. Res. Commun. 526 (2), 497–504. 10.1016/j.bbrc.2020.03.091 32241545

[B2] Bartlett J. J. Trivedi P. C. Pulinilkunnil T. (2017). Autophagic dysregulation in doxorubicin cardiomyopathy. J. Mol. Cell Cardiol. 104, 1–8. 10.1016/j.yjmcc.2017.01.007 28108310

[B3] Chen Y. Shi S. Dai Y. (2022). Research progress of therapeutic drugs for doxorubicin-induced cardiomyopathy. Biomed. Pharmacother. 156, 113903. 10.1016/j.biopha.2022.113903 36279722

[B4] Chen Y. Xu M. Liu X. M. Wang J. X. Sun M. F. Song J. X. (2024). Mechanistic study of huangqi guizhi wuwu decoction amelioration of doxorubicin-induced cardiotoxicity by reducing oxidative stress and inhibiting cellular pyroptosis. Biomed. Pharmacother. 175, 116653. 10.1016/j.biopha.2024.116653 38688172

[B5] Christidi E. Brunham L. R. (2021). Regulated cell death pathways in doxorubicin-induced cardiotoxicity. Cell Death Dis. 12 (4), 339. 10.1038/s41419-021-03614-x 33795647 PMC8017015

[B6] Eneh C. Lekkala M. R. (2024). “Dexrazoxane. 2023 Jul 17,” in StatPearls. (Treasure Island, FL: StatPearls Publishing). 32809394

[B7] Fan D. Jin Z. Cao J. Li Y. He T. Zhang W. (2023). Leucine zipper protein 1 prevents doxorubicin-induced cardiotoxicity in mice. Redox Biol. 64, 102780. 10.1016/j.redox.2023.102780 37354826 PMC10320257

[B8] Fan X. He Y. Wu G. Chen H. Cheng X. Zhan Y. (2023). Sirt3 activates autophagy to prevent DOX-induced senescence by inactivating PI3K/AKT/mTOR pathway in A549 cells. Biochim. Biophys. Acta Mol. Cell Res. 1870 (2), 119411. 10.1016/j.bbamcr.2022.119411 36521686

[B9] Fang Z. Liu Z. Tao B. Jiang X. (2023). Engeletin mediates antiarrhythmic effects in mice with isoproterenol-induced cardiac remodeling. Biomed. Pharmacother. 161, 114439. 10.1016/j.biopha.2023.114439 36848751

[B10] Feng H. He Y. La L. Hou C. Song L. Yang Q. (2020). The flavonoid-enriched extract from the root of smilax China L. inhibits inflammatory responses *via* the TLR-4-mediated signaling pathway. J. Ethnopharmacol. 256, 112785. 10.1016/j.jep.2020.112785 32222576

[B11] Godishala A. Yang S. Asnani A. (2018). Cardioprotection in the modern era of cancer chemotherapy. Cardiol. Rev. 26 (3), 113–121. 10.1097/CRD.0000000000000194 29608498

[B12] Kong C. Y. Guo Z. Song P. Zhang X. Yuan Y. P. Teng T. (2022). Underlying the mechanisms of doxorubicin-induced acute cardiotoxicity: oxidative stress and cell death. Int. J. Biol. Sci. 18 (2), 760–770. 10.7150/ijbs.65258 35002523 PMC8741835

[B13] Kong L. Zhang W. Liu S. Zhong Z. Zheng G. (2022). Quercetin, engelitin and caffeic acid of smilax China L. polyphenols, stimulate 3T3-L1 adipocytes to brown-like adipocytes *via* β3-AR/AMPK signaling pathway. Plant Foods Hum. Nutr. 77 (4), 529–537. 10.1007/s11130-022-00996-x 35986845

[B14] Li B. Yang X. Zhang P. Guo J. Rong K. Wang X. (2022). Engeletin alleviates the inflammation and apoptosis in intervertebral disc degeneration *via* inhibiting the NF-κB and MAPK pathways. J. Inflamm. Res. 15, 5767–5783. 10.2147/JIR.S371809 36238766 PMC9553281

[B15] Liu T. Li Y. Sun J. Tian G. Shi Z. (2020). Engeletin suppresses lung cancer progression by inducing apoptotic cell death through modulating the XIAP signaling pathway: a molecular mechanism involving ER stress. Biomed. Pharmacother. 128, 110221. 10.1016/j.biopha.2020.110221 32447208

[B16] Lu Q. B. Ding Y. Liu Y. Wang Z. C. Wu Y. J. Niu K. M. (2023). Metrnl ameliorates diabetic cardiomyopathy *via* inactivation of cGAS/STING signaling dependent on LKB1/AMPK/ULK1-mediated autophagy. J. Adv. Res. 51, 161–179. 10.1016/j.jare.2022.10.014 36334887 PMC10491969

[B17] Monahan D. S. Flaherty E. Hameed A. Duffy G. P. (2021). Resveratrol significantly improves cell survival in comparison to dexrazoxane and carvedilol in a h9c2 model of doxorubicin induced cardiotoxicity. Biomed. Pharmacother. 140, 111702. 10.1016/j.biopha.2021.111702 34015579

[B18] Pushkala V. P. Sulekha S. M. P. Mathukumar S. Ragavi B. Sowmiya U. (2022). Molecular docking analysis of siddha formulation parangipattai chooranam against vaginal candidiasis. Appl. Biochem. Biotechnol. 194 (3), 1039–1050. 10.1007/s12010-022-03813-y 34997904

[B19] Qiu H. Huang S. Liu Y. Liu L. Guo F. Guo Y. (2024). Idebenone alleviates doxorubicin-induced cardiotoxicity by stabilizing FSP1 to inhibit ferroptosis. Acta Pharm. Sin. B 14 (6), 2581–2597. 10.1016/j.apsb.2024.03.015 38828159 PMC11143507

[B20] Qu Y. Gao R. Wei X. Sun X. Yang K. Shi H. (2022). Gasdermin D mediates endoplasmic reticulum stress *via* FAM134B to regulate cardiomyocyte autophagy and apoptosis in doxorubicin-induced cardiotoxicity published correction appears in cell death dis. 2022 Nov 22;13(11):983. Cell Death Dis. 13 (10), 901. 10.1038/s41419-022-05333-3 36289195 PMC9606128

[B21] Rawat P. S. Jaiswal A. Khurana A. Bhatti J. S. Navik U. (2021). Doxorubicin-induced cardiotoxicity: an update on the molecular mechanism and novel therapeutic strategies for effective management. Biomed. Pharmacother. 139, 111708. 10.1016/j.biopha.2021.111708 34243633

[B22] Sheibani M. Azizi Y. Shayan M. Nezamoleslami S. Eslami F. Farjoo M. H. (2022). Doxorubicin-induced cardiotoxicity: an overview on pre-clinical therapeutic approaches. Cardiovasc. Toxicol. 22 (4), 292–310. 10.1007/s12012-022-09721-1 35061218

[B23] Shi S. Chen Y. Luo Z. Nie G. Dai Y. (2023). Role of oxidative stress and inflammation-related signaling pathways in doxorubicin-induced cardiomyopathy. Cell Commun. Signal 21 (1), 61. 10.1186/s12964-023-01077-5 36918950 PMC10012797

[B24] Singh M. Nicol A. T. DelPozzo J. Wei J. Nguyen T. (2022). Demystifying the relationship between metformin, AMPK, and doxorubicin cardiotoxicity. Front. Cardiovasc. Med. 9, 839644. 10.3389/fcvm.2022.839644 35141304 PMC8818847

[B25] Timm K. N. Tyler D. J. (2020). The role of AMPK activation for cardioprotection in doxorubicin-induced cardiotoxicity. Cardiovasc. Drugs Ther. 34 (2), 255–269. 10.1007/s10557-020-06941-x 32034646 PMC7125062

[B26] Wei J. Zhang Y. Li D. Xie T. Li J. (2020). Integrating network pharmacology and component analysis study on anti-atherosclerotic mechanisms of total flavonoids of Engelhardia roxburghiana leaves in mice. Chem. Biodivers. 17 (3), e1900629. 10.1002/cbdv.201900629 31943763

[B27] Wu B. B. Leung K. T. Poon E. N. (2022). Mitochondrial-targeted therapy for doxorubicin-induced cardiotoxicity. Int. J. Mol. Sci. 23 (3), 1912. 10.3390/ijms23031912 35163838 PMC8837080

[B28] Wu L. Du Y. Wang L. Zhang Y. Ren J. (2024). Inhibition of METTL3 ameliorates doxorubicin-induced cardiotoxicity through suppression of TFRC-mediated ferroptosis. Redox Biol. 72, 103157. 10.1016/j.redox.2024.103157 38631119 PMC11033199

[B29] Xu Y. Zhang J. Gao F. Cheng W. Wei C. (2023). Engeletin alleviates cerebral ischemia reperfusion-induced neuroinflammation *via* the HMGB1/TLR4/NF-κB network. J. Cell Mol. Med. 27 (12), 1653–1663. 10.1111/jcmm.17758 37132060 PMC10273068

[B30] Zhang W. Wang X. Tang Y. Huang C. (2023). Melatonin alleviates doxorubicin-induced cardiotoxicity *via* inhibiting oxidative stress, pyroptosis and apoptosis by activating Sirt1/Nrf2 pathway. Biomed. Pharmacother. 162, 114591. 10.1016/j.biopha.2023.114591 36965257

[B31] Zhao X. Chen R. Shi Y. Zhang X. Tian C. Xia D. (2020). Antioxidant and anti-inflammatory activities of six flavonoids from Smilax glabra roxb. Molecules 25 (22), 5295. 10.3390/molecules25225295 33202848 PMC7697956

[B32] Zhong Z. Gao Y. Zhou J. Wang F. Zhang P. Hu S. (2023). Inhibiting mir-34a-5p regulates doxorubicin-induced autophagy disorder and alleviates myocardial pyroptosis by targeting Sirt3-AMPK pathway. Biomed. Pharmacother. 168, 115654. 10.1016/j.biopha.2023.115654 37806095

[B33] Zhong X. Huang R. Chen X. Lei Y. (2023). A review on the pharmacological aspects of engeletin as natural compound. Drug Des. Devel Ther. 17, 3833–3843. 10.2147/DDDT.S437703 38152488 PMC10752015

[B34] Zhou B. Zhang J. Chen Y. Liu Y. Tang X. Xia P. (2022). Puerarin protects against sepsis-induced myocardial injury through AMPK-mediated ferroptosis signaling. Aging (Albany NY) 14 (8), 3617–3632. 10.18632/aging.204033 35482440 PMC9085223

[B35] Zhuo C. Xin J. Huang W. Zhang D. Yan X. (2023). Irisin protects against doxorubicin-induced cardiotoxicity by improving AMPK-Nrf2 dependent mitochondrial fusion and strengthening endogenous anti-oxidant defense mechanisms. Toxicology 494, 153597. 10.1016/j.tox.2023.153597 37499777

