# Supplementary material for: Engeletin alleviates doxorubicin-induced cardiotoxicity via the AMPK pathway in mice
Source: Front Pharmacol. 2026 Feb 26;17:1741741. doi: 10.3389/fphar.2026.1741741 (PMC12979110; doi:10.3389/fphar.2026.1741741)

Figure-2C

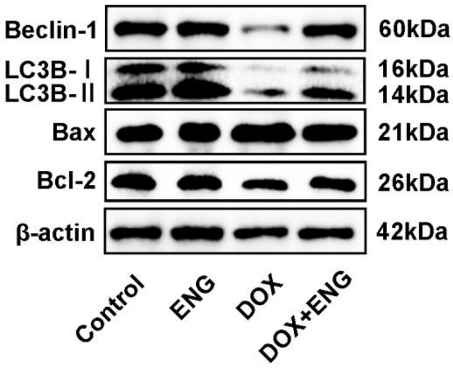

Beclin

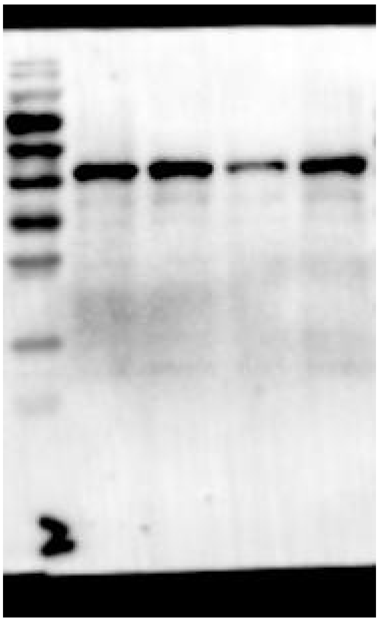

LC3B

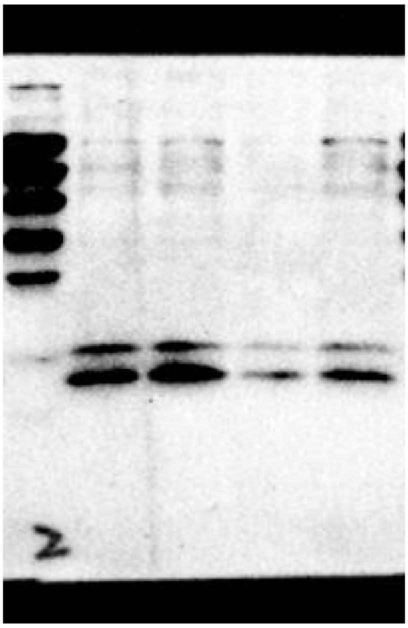

BAX

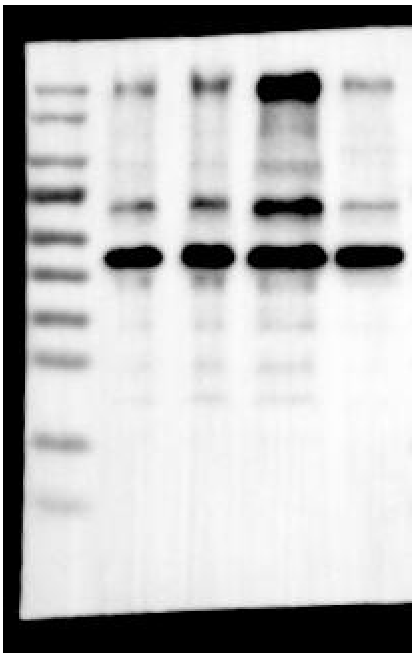

BCL2

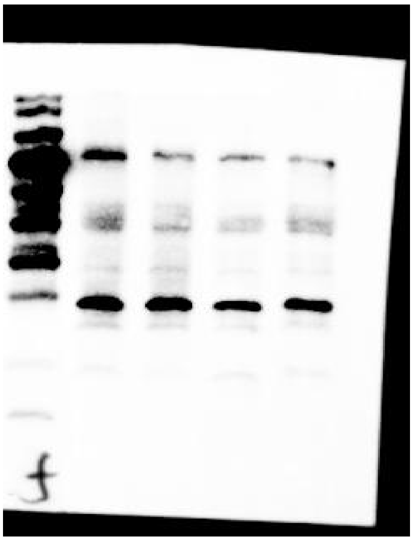

Actin

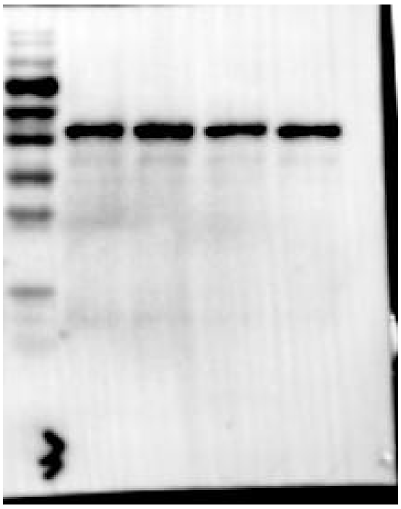

Figure-4D

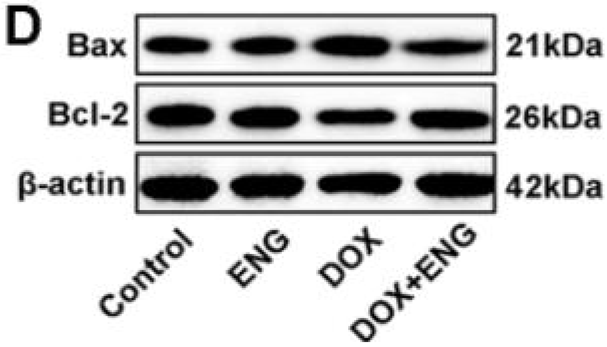

BAX

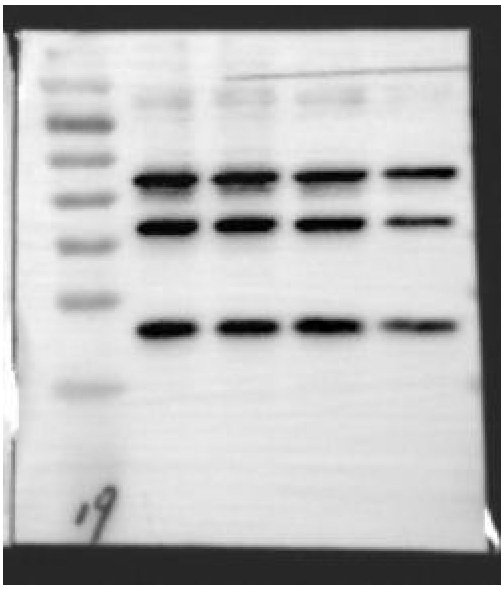

BCL-2

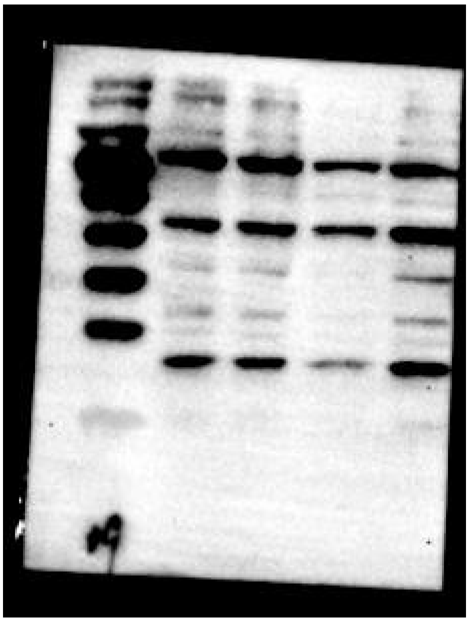

Actin

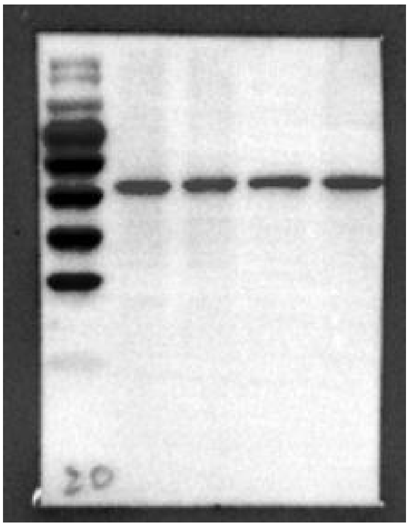

Figure-4H

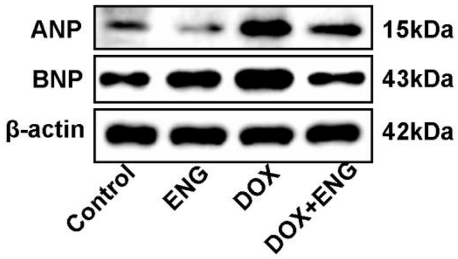

ANP

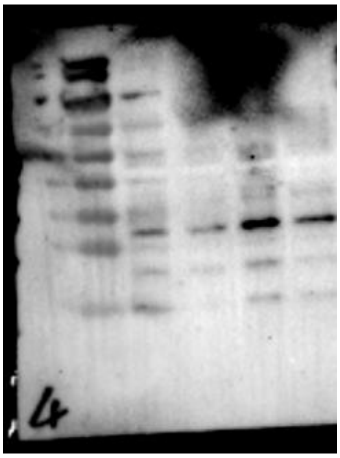

BNP

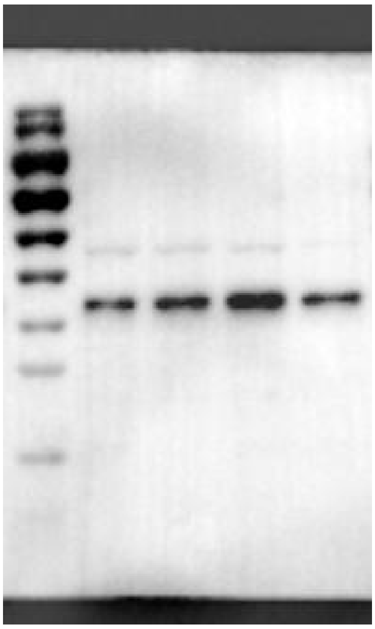

Actin

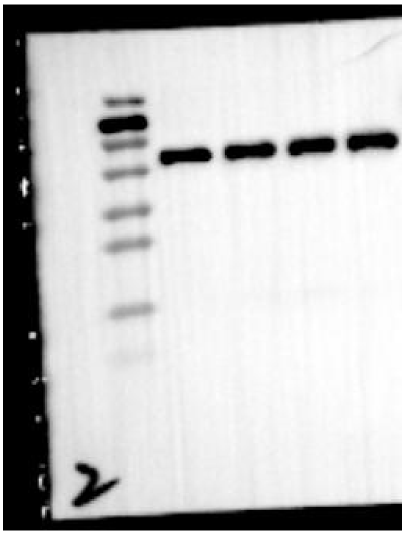

Figure-5D

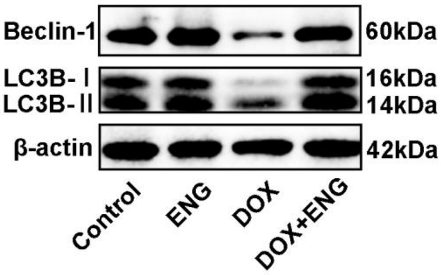

Beclin

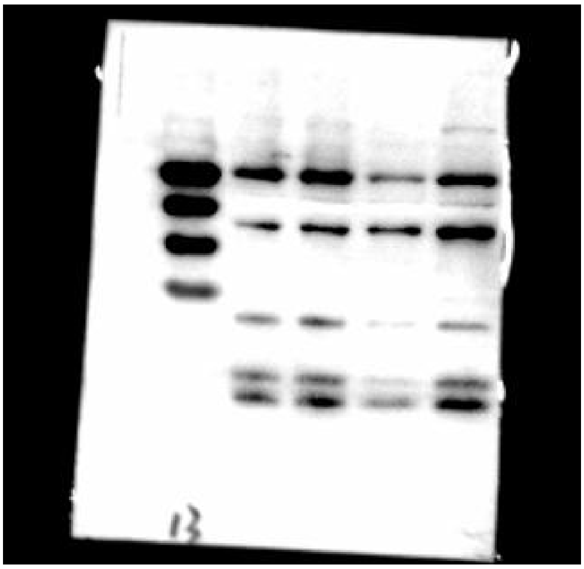

LC3B

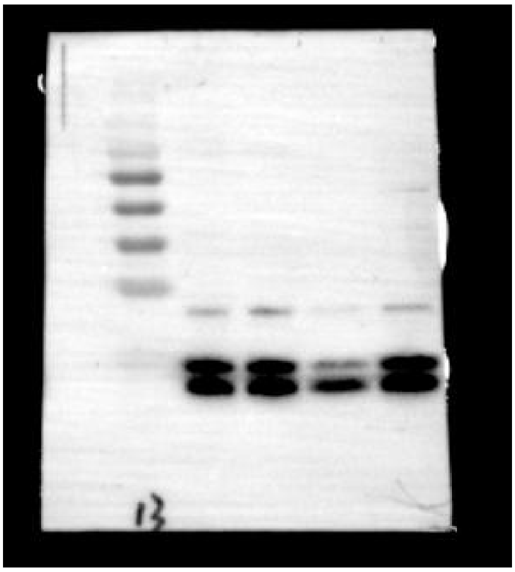

Actin

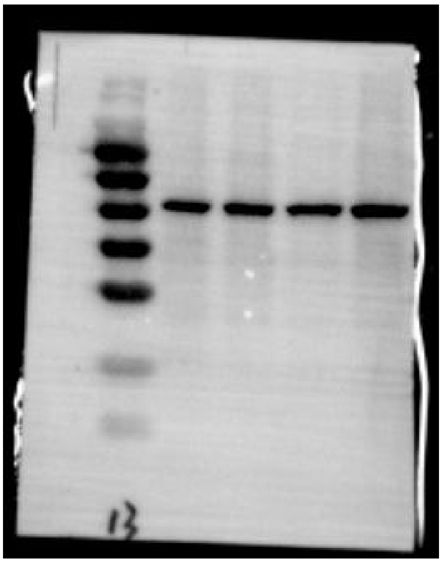

Figure-6A

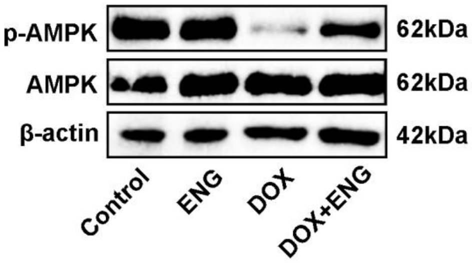

Pampk

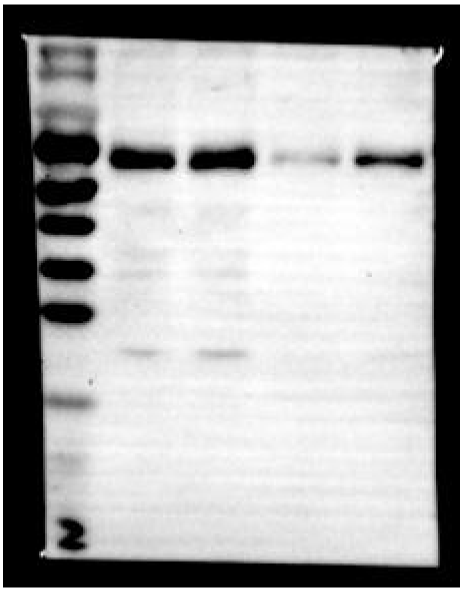

AMPK

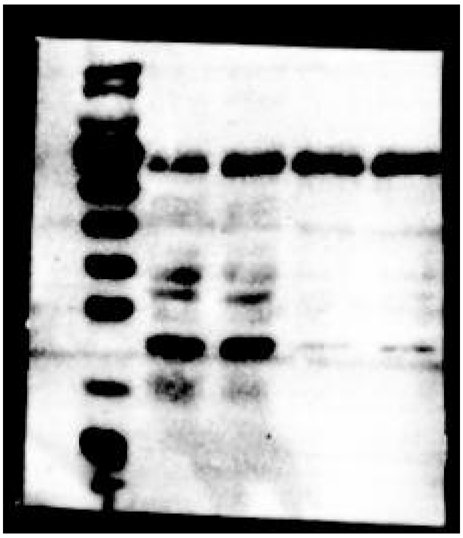

Actin

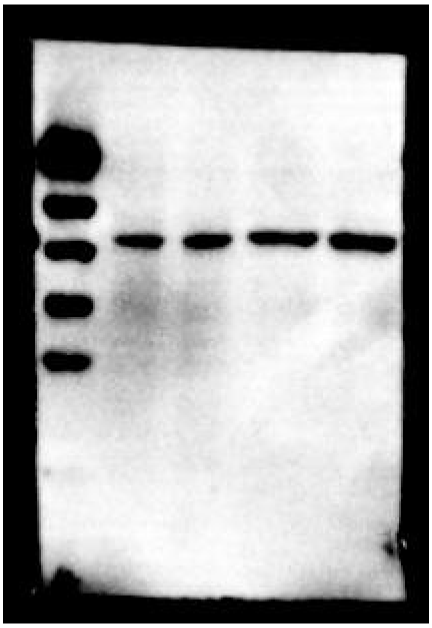

Figure-6C

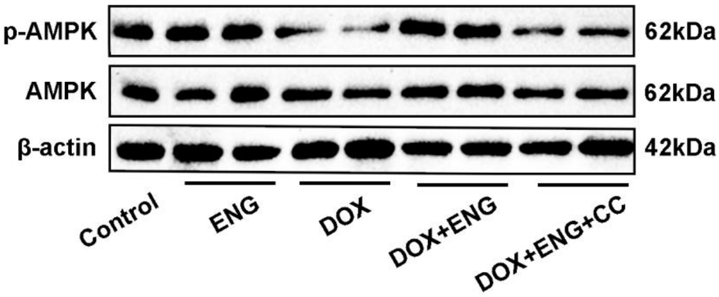

pAMPK

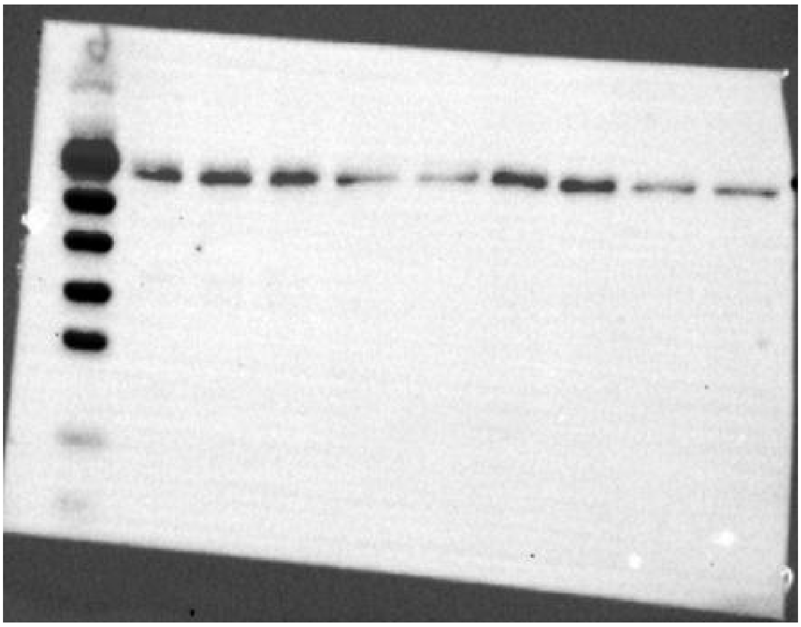

AMPK

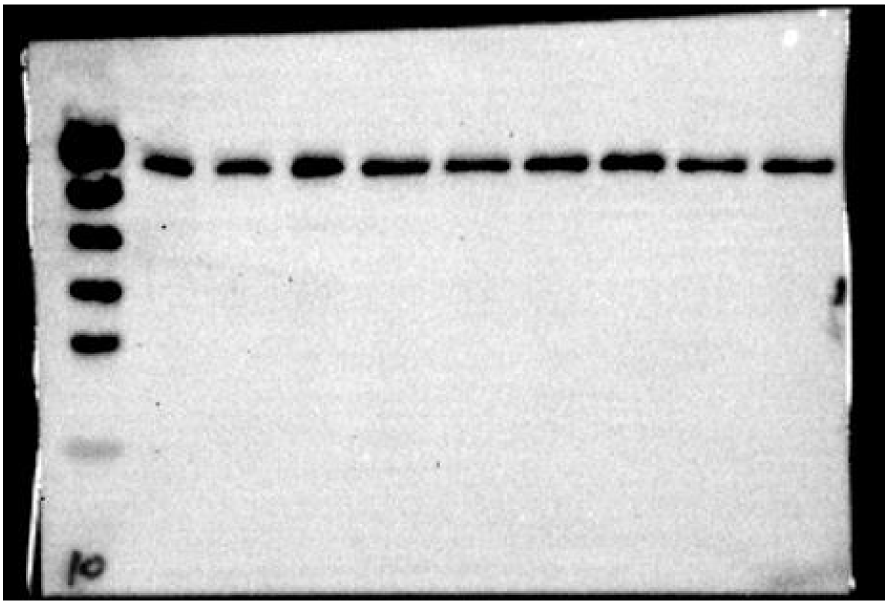

Actin

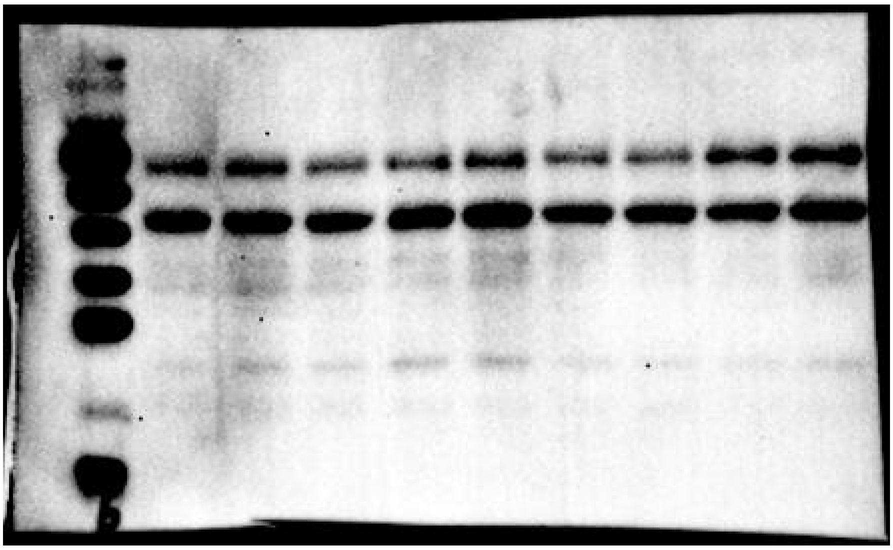

Figure-6E

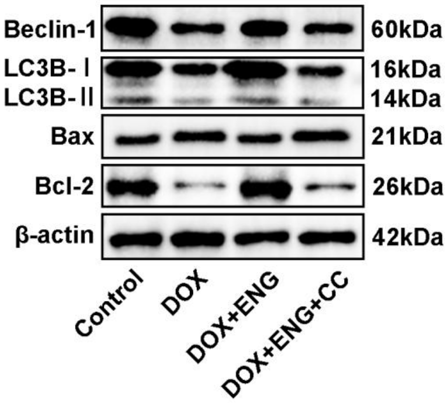

Beclin

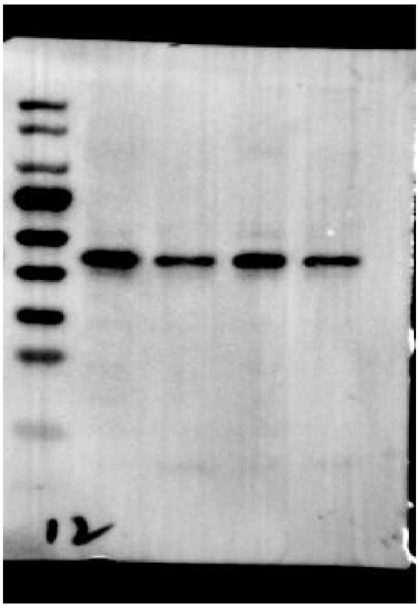

LC3B

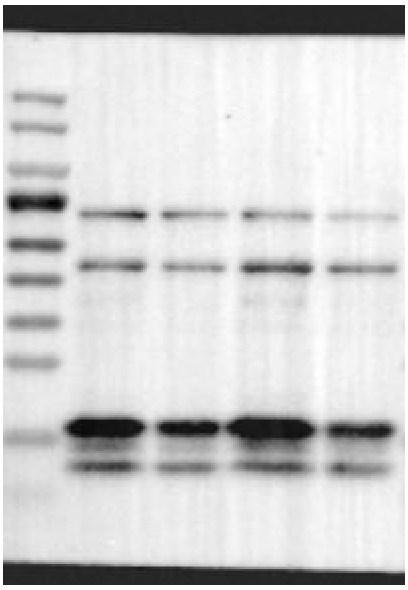

BAX

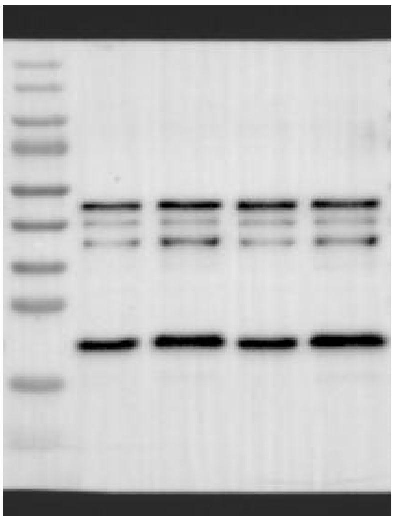

BCL2

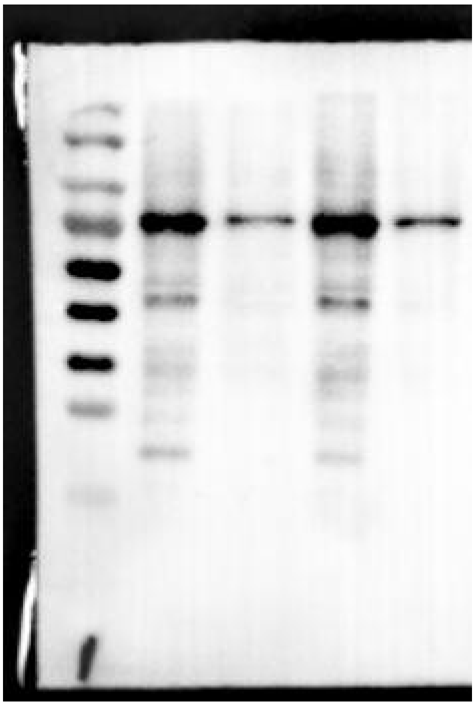

Actin

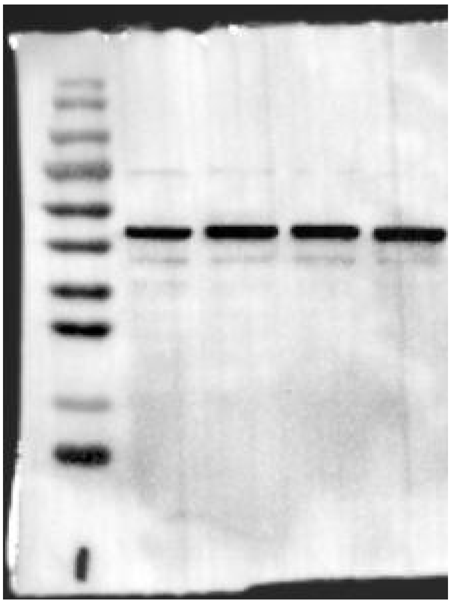

Supplement: Supplementary file 1 [file DataSheet1.pdf]
